# Distribution and Characterization of Progenitor Cells within the Human Filum Terminale

**DOI:** 10.1371/journal.pone.0027393

**Published:** 2011-11-11

**Authors:** Lisa Arvidsson, Michael Fagerlund, Nasren Jaff, Amina Ossoinak, Katarina Jansson, Anders Hägerstrand, Clas B. Johansson, Lou Brundin, Mikael Svensson

**Affiliations:** 1 Department of Clinical Neuroscience, Dept of Neurosurgery and Neurology, Karolinska Institutet, Karolinska University Hospital, Stockholm, Sweden; 2 NeuroNova, Stockholm, Sweden; 3 Center for Molecular Medicine, Department of Clinical Neuroscience, Karolinska Institutet, Stockholm, Sweden; Université Pierre et Marie Curie-Paris6, INSERM, CNRS, France

## Abstract

**Background:**

Filum terminale (FT) is a structure that is intimately associated with conus medullaris, the most caudal part of the spinal cord. It is well documented that certain regions of the adult human central nervous system contains undifferentiated, progenitor cells or multipotent precursors. The primary objective of this study was to describe the distribution and progenitor features of this cell population in humans, and to confirm their ability to differentiate within the neuroectodermal lineage.

**Methodology/Principal Findings:**

We demonstrate that neural stem/progenitor cells are present in FT obtained from patients treated for tethered cord. When human or rat FT-derived cells were cultured in defined medium, they proliferated and formed neurospheres in 13 out of 21 individuals. Cells expressing Sox2 and Musashi-1 were found to outline the central canal, and also to be distributed in islets throughout the whole FT. Following plating, the cells developed antigen profiles characteristic of astrocytes (GFAP) and neurons (β-III-tubulin). Addition of PDGF-BB directed the cells towards a neuronal fate. Moreover, the cells obtained from young donors shows higher capacity for proliferation and are easier to expand than cells derived from older donors.

**Conclusion/Significance:**

The identification of *bona fide* neural progenitor cells in FT suggests a possible role for progenitor cells in this extension of conus medullaris and may provide an additional source of such cells for possible therapeutic purposes.

Filum terminale, human, progenitor cells, neuron, astrocytes, spinal cord.

## Introduction

Filum terminale (FT) is a structure that during development and is attached to the first segment of the coccyx. Under normal conditions the FT is thin and does not prevent free movements of the spinal cord. In few individuals this connection is maintained which then effects growth and posture, induces pain and leads to neurological symptoms of the condition teathered cord. The normal human FT is mainly a fibrous band, composed of two segments, one intradural and one extradural. FT contains an extension of the central canal which is lined with ciliated ependymal cells [1], [2], [3], [4], [5], [6], [7]. Throughout the FT this canal can disappear and reappear in distal portions. To avoid neurological deficit, FT is divided during surgery in patients that suffer from tethered cord [4], [8]. This surgery can be undertaken with a small resection of FT without development of neurological deficits. With ethical permission we utilized these surgical procedures to harvest tissue from FT.

Early reports suggested that FT could contain neural tissue [9], [10]. Choi et al. demonstrated that FT contains an abundance of glial cells, ependymal cells and what has been described as degenerated neuronal elements [1]. It was recently shown that cells from FT contain progenitor–like cells, which could form neurospheres. These neurosphere-derived cells could differentiate into neurons, astrocytes and oligodendrocytes [11]. Since the original published article on *in vitro* isolation of neural stem cells in the adult central nervous system (CNS), several groups have shown that stem/progenitor cells cultured from the ventricular wall of adult humans may differentiate into neurons [12]. These cells can develop mature biological and electrophysiological features, including synaptic communication [13], [14]. These findings opened the possibility to replace lost neurons or glia by transplantation of cultured neural progenitor cells harvested and multiplied from the ventricular wall.

In this study we investigate the occurrence and distribution of neural stem/progenitor cells in FT. We explored the expression of stem cell markers; reveal the distribution of progenitor cells and their capacity for expansion and response to growth factor stimulation. We applied protocols previously used for characterization of progenitors [12] already known to be present in the subventricular zone (SVZ) and in the dentate gyrus of the hippocampal formation [15], [16], [17], [18], [19]. Here we describe the localization and morphology of cells expressing progenitor cell characteristics and provide further evidence for the existence of a progenitor cell pool in the human FT.

## Results

### The distribution of progenitor cells in the filum terminale

**Figure 1:**
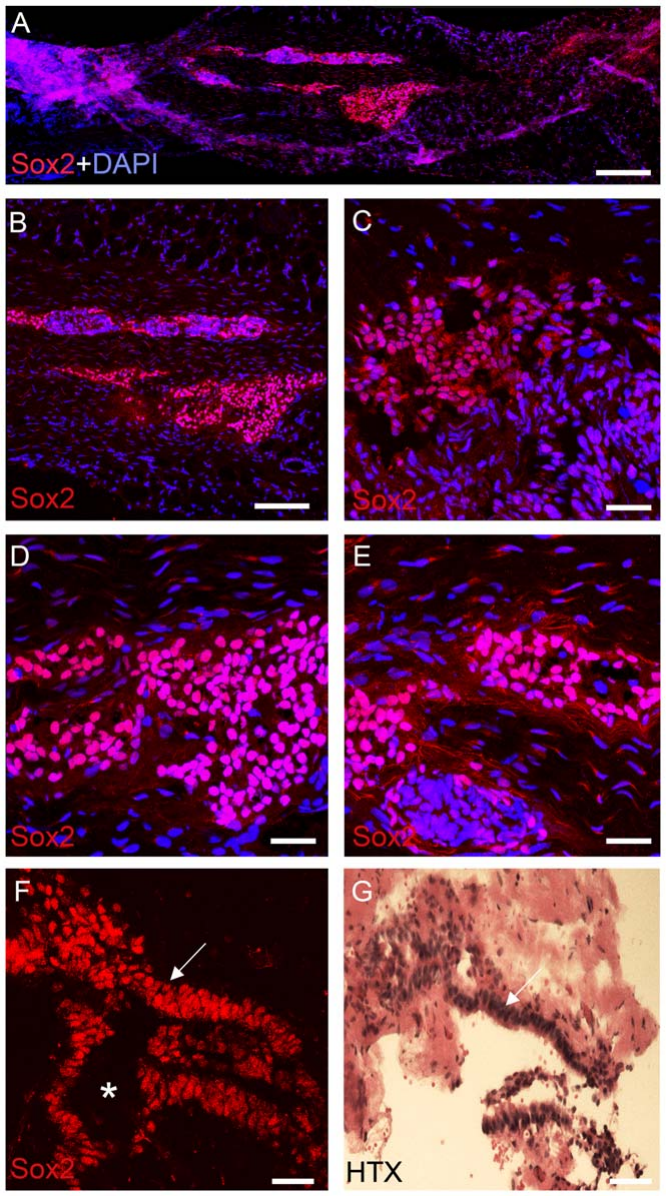
Distribution of Sox2-positive cells within filum terminale. (A) Longitudinal section represents an overview of the distribution and localization of the neuronal progenitor cells in human FT revealed by Sox2-positive cells (red) and the nuclear staining DAPI (blue). (B–E) Represent higher magnifications demonstrating Sox2-positive cells localized subependymally and outlining the central canal. (F and G) represent coronal adjacent sections, (F) stained with florescence staining. The central canal is indicated with an asterix (G) Coronar section stained with Haematoxylin–eosin staining. Arrows (in F and G) indicates corresponding areas. A–F; confocal, G; light microscopy. Scale bars: A = 100 µm; B = 50 µm; C−G = 40 µm.

In order to identify the cellular elements and localize progenitor cells in human FT, immunohistochemistry was performed using the progenitor cell markers Sox2 and Musashi-1 on sagittal and /or coronal sections. Sox2-immunoreative cells were abundant in the FT (Fig. 1). Subependymal bands of 10–20 cells appeared in streaks and small clusters, we also found very large clusters of more than 500 cells/section (Fig.1 C, D and E). Sox-2 labelled cells were found in the ependymal cell layers surrounding the central canal. (Fig. 1 F, G). There was no apparent rostrocaudal gradient of Sox2-immunoreactive cell density.

**Figure 2:**
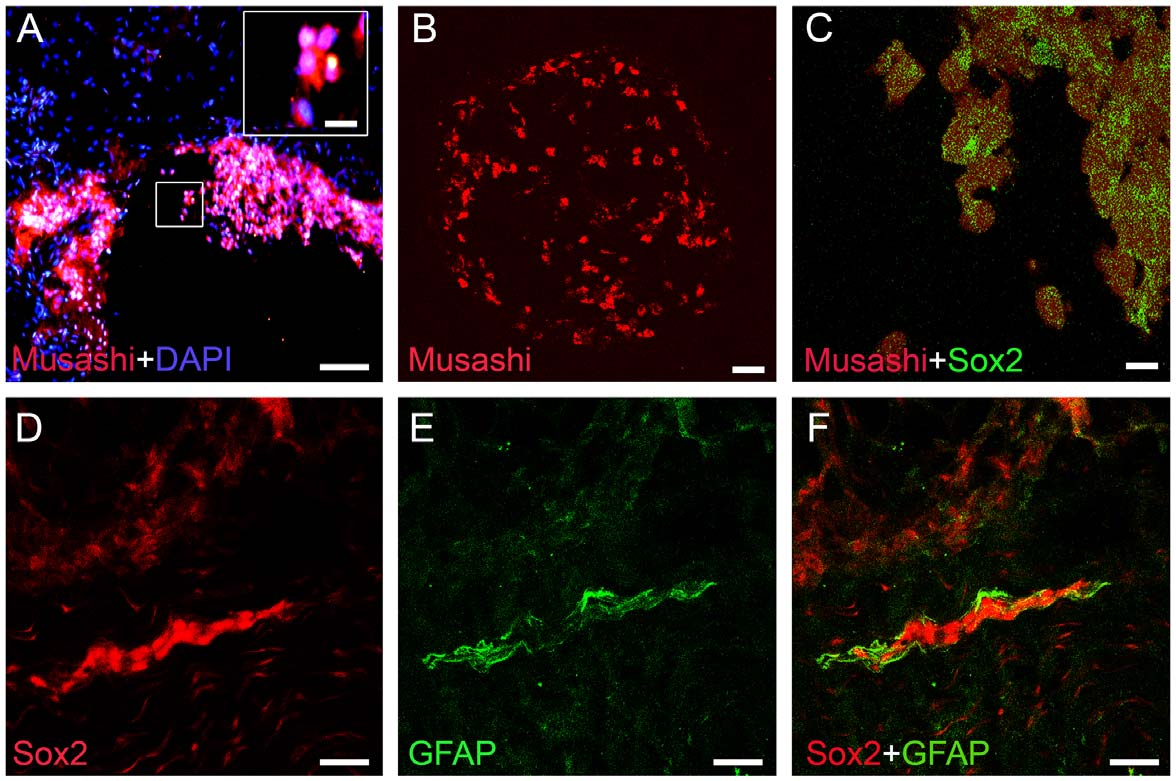
Presence of additional progenitor markers in the filum terminale. (A) Longitudinal section and (B) coronal section shows clusters of Musashi-positive cells within the FT (red) and nucleus staining DAPI (blue). (A) A large cluster of Musashi-positive cells at the surface of the FT. Insert represents higher magnification of the boxed area. (B) Small clusters of Musashi-positive cells within the FT. (C–F) Double-immunolabeled sections from human FT show that Sox2 immunoreactivity (green) was colocalized with Musashi immunoreactivity (red) (C). The Sox2 (red) cells were also expressing GFAP (green) (D–F). Scale bars: A,D,E,F = 40 μm, B,C and insert box = 20 μm.

Musashi-1 immunoreactivity was also detected in ependymal cells surrounding the central canal as well as in the subependymal compartment where the cells were found in organized layers but also scattered in larger clusters at the surface of FT (Fig. 2A). Musashi-immunoreactive cells could also be found in smaller islets at a distance from the central canal (Fig. 2B **).** Double immunolabelling revealed that Musashi-1-positive cells were also Sox2-positive (Fig. 2C). A very strong GFAP immunoreactivity was observed in the progenitor cells, with a weaker GFAP immunoreactivity in the surrounding tissue (Fig. 2D, E). Cells expressing strong immunolabelling for Sox2 were also GFAP positive (Fig. 2E, F). As determined from β-III tubulin staining, the axonal density of the FT was very low, and neuronal cell bodies were rarely found (NeuN, data not shown).

### Expansion and differentiation of progenitor cells from filum terminale

**Figure 3:**
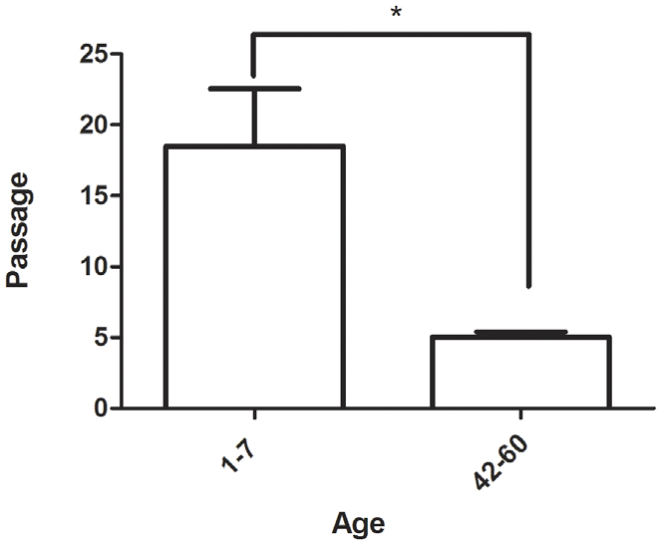
Quantitative determination of cell expansion from young donors (1–7years) and old donors (42–60years). The number of obtainable cell passages in young donors was significantly higher than old donors. *P <0.05.

**Figure 4:**
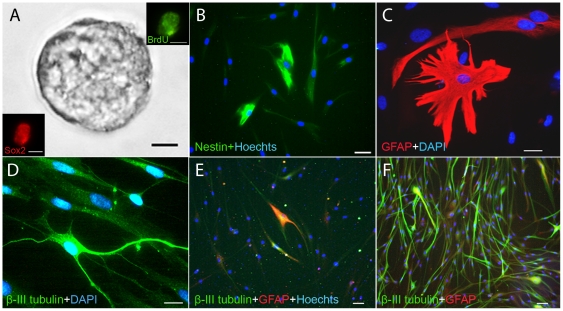
Human FT cells in culture. (A) Neurosphere after second passage. BrdU-positive (green) and Sox2-positive (red) cells in neurosphere from human FT (Insert in A). (B) Nestin-positive cells (green) after 28 days with nuclear marker Hoechst (blue). (C) GFAP-positive cells with astroglial shape (red) and (D) One neuronal (β-III-tubulin) cell as it appears after 11 days of differentiation (blue = DAPI). (E) Expansion of cells without PDGF-BB. (F) Cells expanded in culture medium exposed to PDGF-BB for 8 days, GFAP (red), β-III tubulin (green) and nuclear marker Hoechst (blue). Bars: A and B = 10 μm, Insert = 25 μm, C and D = 5 μm and E−F = 20 μm.

We next extended our study to learn whether we could isolate and expand neural stem/progenitor cells from FT. Specimens from patients of different ages (1–60 years) were successfully isolated and similar to the cells isolated from other human brain tissues, SVZ tissue, single cells were able to form neurosphere in 13 out of 21 patients (62%, Table 1). FT derived neurospheres were frequently detected from young patients and had an excellent growth capacity and could be passaged for up to 15–30 passages (n = 6). Moreover, these progenitor cells shows higher capacity for proliferation and is easier to expand than cells derived from older donors. However, it was also possible to propagate cells from older donors but with lower growth rate compared to the younger donors (Table 1, Fig.3). These spheres resembled spheres obtained from the previously described regenerative zones of the CNS (Fig. 4A). Proliferation of the progenitor cells was confirmed by BrdU incorporation and Sox2 staining (insert Fig. 4A). To test if FT derived neurospheres have self-renewal capacity, we isolated single cells from the neurospheres and cultured them at a density of 60 cells per ml. Single isolated cells gave rise to secondary neurospheres, demonstrating that some cells possess self-renewal capacity. A clonal expansion study showed that 60 primary culture cells was the minimal number needed to form one neurosphere which indicate a relatively strong self-renewal capacity. The expansion rate and yield of cells was highly variable between individuals. In spite of this, the cells could be cultured for up to 29 passages**.** In cells at early stages of differentiation nestin immunolabeling was present (Fig. 4B).

**Table 1:** Age of donors and the number of neurospheres formed.

Age group	n	Spheres	No spheres
1–5	8	6	2
6–15	8	4	4
>30	5	3	2

Eleven days post-differentiation, we observed GFAP-positive cells with morphological and phenotypic characteristics of astroglia (Fig. 4C). Under regular growth conditions FT cultures 5% of the cells expressed β-III-tubulin upon differentiation (Fig. 4D) In a subset of patients the β-III-tubulin expression was validated for each expansion of the cells and was maintained up to the maximum of 29 expansions (Table 2). Cells of the oligodendrocyte lineage were not found (data not shown). Concomitant with differentiation, the number of nestin-positive cells declined, as expected. PDGF-BB was added to the differentiation medium since it acts as a mitogen in the early phases of stem cell differentiation and promotes survival and proliferation of immature neurons [20], [21], [22]. Administration of PDGF-BB resulted in a significant (ten fold) increase in the amount of β-III-tubulin positive cells, in comparison to control cultures (Fig. 4E, F).

**Table 2:** The longitudinal expression of β-III-tubulin followed in a subset of patients.

Age	Gender	Expansions	β-III-tubulin
1	F	P15	+
2	M	P20	+
4	F	P29	+
7	M	P10	+
42	M	P6	+
60	M	P5	not done

### Comparisons to rat filum terminale

**Figure 5:**
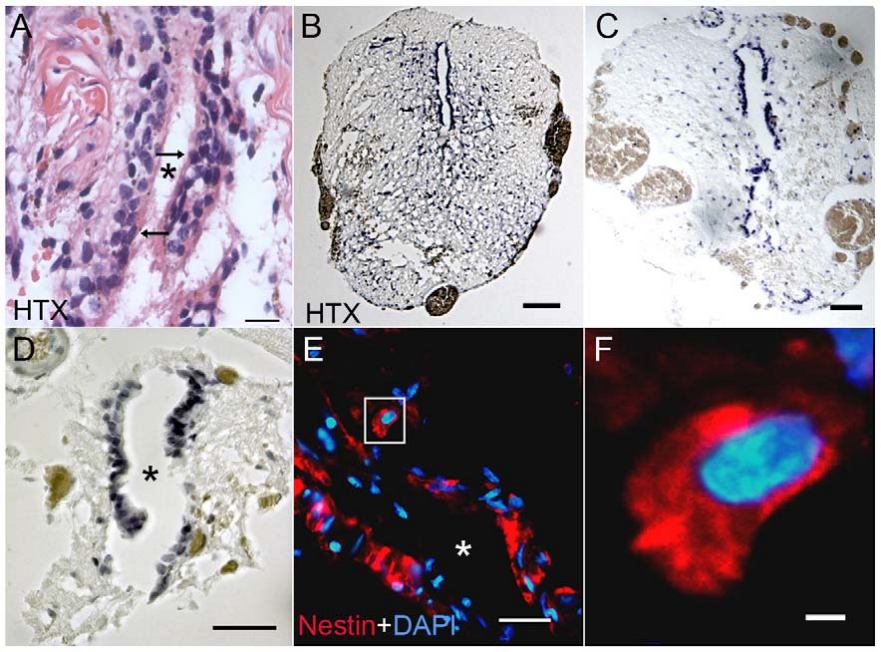
Anatomical comparison between the human and rat filum terminale. (A) Longitudinal section of human FT stained with haematoxylin-eosin. The human FT mostly consists of astrocytes and ependymal cells (see arrows) (B–D). Transverse sections of rat FT stepwise from conus (B) and further caudally (C and D) the central canal is prominent in the middle of the rat film. Rounded brown structures in the perperiphery represent nerve roots. (E and F). Longitudinal sections of the rat FT labelled with a nestin antibody (red) and a nuclear marker DAPI (blue). (F) Higher magnification of boxed area in (E). Bars: A and E = 40 µm, B and C = 100 µm, F = 5 µm. Asterix =  central canal.

Longitudinal sections from human and rat FT confirmed the presence of a central canal structure with a border of ependymal cells within the vascular fibrous and fatty tissue and collagen bands that constitutes the FT. In the rat the central canal is large and well defined whereas in the human, it is more of a scattered system of tubular structures lined by ependymal-type cells (Fig. 5A). These structures were considered to represent embryonic ependymal remnants in the FT. Similar central canal structures with ependymal cells were found at the level of conus medullaris of rat (Fig. 5B) and was prominent at more caudal levels (Fig. 5C and D). Also in the rat FT, the ependymal cells expressed nestin, a marker for progenitor cells in the CNS (Fig. 5E,F). For comparison, rat FT cells were also cultured. We obtained neurospheres from 5 out of 13 cultures (39%). Cells from rat FT were difficult to expand, which may relate to the small amounts of harvested tissue.

## Discussion

The caudal part of the spinal cord in reptiles, mice and avians are different. Reptiles lack a true FT, whereas in birds, the equivalent region of FT contains neurons [23]. In contrast, such is not the case for mouse in which no neural markers are present in this region [24]. The present morphological study of the human FT was carried out since this region has been shown to contain cells with stem cell properties [11]. Here we focused on the location, distribution and organization of the progenitor cells within the FT. We found that Sox2 positive cells were abundant in the ependymal layer and the subependymal area adjacent to the central canal as well as in the form of islets of Musashi positive cells. The Musashi family belongs to a conserved group of neuronal RNA-binding proteins [25]. Sox2 is a transcription factor which is critical in maintaining pluripotency and self-renewal. Sox2 and Musashi have been shown to be selectively expressed in the neuronal progenitor cells [25], [26], [27], [28]. Co-localization of Musashi and Sox2 was seen in a majority of cells in the FT. The general morphology of the human FT has previously been described [1] and our findings are in line with the previous findings, showing the presence of fibrous tissue, astrocytes and ependymal cells. We were, however, surprised by finding so many cells expressing pluripotency markers and that they were present throughout the length of FT.

The establishment of viable cultures was not always achieved, this could, in part be explained by the operating technique which often involves electro-coagulation, and thereby may devitalize the cells. The yield of cells was highly variable between individuals which could be explained by the variation in anatomy between individuals together with the use of electro-coagulation during the surgical procedure. These patients are operated for tethered cord syndrome which is a pathological state. The tissue decribed here is thereby not entirely normal, although tumors are excluded. For ethical reasons healthy filum is not accessible. This rare procedure is performed only 10–15 times per year and some material was discarded due to the finding of a tumor, such as lipoma or ependymoma which may arise in this region[29], [30].

Our findings are interesting from two clinical perspectives; as source of progenitor cells and from the perspective of tumor formation, which was not addressed in this study. We confirmed here that FT contains neural stem/progenitor cells. Since all humans possess this structure, these cells can potentially be used for auto-transplantation purposes. Identification of neural stem cells in humans has opened possibilities for transplantation strategies to treat neurodegenerative diseases; including supporting compromised neurons in a damaged spinal cord. Neural stem cells are almost impossible to remove without causing permanent damage to the CNS. An alternative structure in the CNS, such as the FT, would give new possibilities to isolate neural stem/progenitor cells without damaging the CNS. Our results revealed that FT derived cells were found to have self-renewal capacity and the ability to grow *in vitro* to form numerous neurospheres in a similar manner as stem/progenitor cells derived from the subventricular zone (SVZ) [12], [14], [31]. Interestingly, the self-renewing properties were maintained also in tissue from adult donors (>age 30), suggesting that this progenitor pool is maintained during adulthood. The result showed also that the progenitor cells detected can reach different cell passage depending on age. The cells from young patients have larger growth capacity, higher proliferative ability and easier to expand than cells derived from older donors. In rat neurosphere proliferation rate, telomerase activity decreases with age [32], also in the hippcampus of rat the proliferation of the CA1 cells decreases with age [33].

After differentiation the cultures were labelled with β-III-tubulin as a marker for neurons [34], [35]. Interestingly, in response to PDGF-BB stimulation, the number of β-III-tubulin positive cells increased. It has been shown that PDGF-BB supports neuronal differentiation of **CNS** stem cells by acting as a mitogen in the early phases of stem cell differentiation, promoting survival and proliferation of immature neurons [20], [21], [22]. The response to PDGF-BB indicates that the cells are early progenitors the cell fate of which can be influenced and directed as is the case for SVZ progenitor cells. Varghese et al., (2009), transplanted human FT cells to Sprague Dawley rats in a global ischemia model and found that the cells developed into GFAP expressing cells, however -as expected, eliciting a strong immune reaction [11]. The abundance of progenitor cells, expressing markers of immaturity throughout the FT may also be of relevance for tumor formation in this region. We speculate that dedifferention occurring in the ependymal progenitor clusters could be the origin of the myxopapillary ependymoma sometimes encountered in the region [29], [36] **.**

In summary, our findings add further evidence for the existence of cells with neuronal progenitor nature within the FT. We demonstrate the abundance and distribution of the cells and their ability to respond to growth factors. We also demonstrate that the progenitor cells detected from young patients have greater growth capacity, higher proliferative ability and more easy to expand than cells derived from older donors. Since the FT can be removed without functional impairment it may represent an attractive CNS reservoir of neural stem/progenitor cells.

## Materials and Methods

### Tissue preparation

#### I. Human tissue

Patients suffering from tethered cord are usually treated surgically by dividing and resecting a part of the FT in the lumbosacral region. This tissue was obtained from young and adult patients (ages 1–60 yrs) between 2006–2011. All patients (n = 21) underwent MRI scans to exclude tumors and were screened for the presence of infectious disease. The tissue was immediately stored in L15 medium (4°C) at the operating theatre and immediately transported to our laboratory. Part of FT tissue from patients was fixed in ice-cold 4% PFA (24 h), and cryoprotected in 17% sucrose before being imbedded in Cryomount (HistoLab Gothenburg). FT was longitudinally and/or coronally sectioned. Another part was performed for the cell culture.

#### II. Rat tissue

Adult male Sprague-Dawley rats (n = 7) (Scanbur B&K, Sollentuna, Sweden), weight 400–500 g, were deeply anesthetized using intraperitoneal injection of 0.5 mg/kg medetomidine (Domitor Vet. 1 mg/ml, OrionPharma) and 75 mg/kg ketamine (Ketalar 50 mg/ml, Pfizer). Animals were sacrificed by transcardial perfusion with body-temperature (37°C) saline followed by cold (4°C) 4% PFA (Apoteket) in PBS. The FT was removed and post fixed for 1 h in cold (4°C) 4% PFA in PBS followed by rinse in PBS and cryoprotected for 1 h in 17% sucrose (w/v) in PBS. FT was cryosectioned in longitudinal and transverse sections (10 µm thick) using a Leica CM3000 (Leica Microsystems). Sections were mounted on SuperFrost Plus microscope slides (Menzel-Gläser).

### Immunohistochemistry

#### 1. Human sections

The sections were washed in PBS for 30 min and then incubated for 60 min in PBS

Containing 1% bovine serum albumin (BSA), 0.3% Triton-X and 0.1% sodium azide.

Sections were incubated with the following antibodies: rabbit anti-Sox2 (Millipore, dil. 1∶200), rabbit anti-Musashi 1 (Millipore, dil. 1∶200), mouse anti-β-III-tubulin (Millipore, dil. 1∶100), mouse anti-NeuN (Millipore, dil. 1∶100), mouse anti-GFAP (Chemicon, dil. 1∶1000), mouse anti-O4 (Chemicon, dil. 1∶50) and rabbit anti-p53 (Santa cruz, dil. 1∶400). All sections were incubated with the primary antibody for 24 h at 4°C, rinsed in PBS and subsequently incubated with species-specific secondary antibodies conjugated with Cy3 (goat anti-mouse, donkey anti-mouse, goat ant-rabbit or donkey anti-rabbit) (Jackson ImmunoResearch) (1∶500) and Alexa 488 donkey anti-rabbit (1∶500) (Invitrogen). Sections were also counterstained with the nuclear marker DAPI (Invitrogen, dil. 1∶2000) or TO-PRO-3 (Invitrogen, dil.1∶10000). Sections were mounted in Mowiol (Calbiochem). For negative controls the primary antibody was omitted (data not shown). For the Avidin Biotin Complex (ABC)-technique sections were incubated with biotinylated secondary antibodies (Vector Laboratories) (1∶200) for 1 h at room temperature, rinsed in PBS and incubated with ABC (Vectastain ABC Kit) for 1 h. After another step of rinsing in PBS followed by TRIS-hydrogen chloride buffer (0.1 M, pH 7.45) immunoreactivity was revealed by incubation in 3, 3′-diaminobenzidine (DAB) by using the DAB Substrate Kit for Peroxidase (Vector Laboratories) for 2–10 min. Sections were rinsed in TRIS and dehydrated through a series of rinses with increasing strength of ethanols to pure xylene and mounted in a non-aqueous DPX-medium.

#### 2. Rat sections

Cryosections from rat FT were air-dried, washed in PBS for 30 min and then incubated for 60 min in PBS containing 1% BSA, 0.3% Triton-X and 0.1% sodium azide to prevent nonspecific binding. All of the primary antibodies used were diluted in this solution. Cell cultures were fixed with 4% PFA in PBS. The cells were blocked in PBS, 0.1% saponin and 5% goat serum. Preparations were incubated with the primary antibody mouse anti-Nestin (dil. 1∶100, Chemicon) for 24 h at 4° C, rinsed in PBS and subsequently incubated with species-specific secondary antibodies diluted in PBS; Alexa 488 donkey anti-mouse 1∶500 (Molecular Probes/Invitrogen). Sections were also counterstained with nuclear markers DAPI (Molecular Probes, Invitrogen). Tissue was also stained for hematoxylin-eosin. The tissue was mounted in Mowiol (Calbiochem, VWR International).

### Microscopy

For analysis of the different antibodies a combination of a laser scanning confocal microscope (Leica TCS SPII) (Leica Microsystems) and a wide field microscope (Leica DM 400B) (Leica Microsystems) was used. The confocal immunofluorescence images were obtained using a 20x (N/A 0.7) and 63x (N/A 1.40) objective. Alexa 488, Cy3 and Cy5 were excited at 488 nm, 543 nm and 633 nm respectively and detected with a 490–520 nm, 560–630 nm and 640–750 nm band-pass filter respectively. Each optical section (1 µm) was averaged four times; images were the projection of 25 successive optical sections into one image. In the fluorescence wide field microscope images were acquired with a Leica DFC320 (Leica) camera. Images of neurospheres in cell culture were taken before and after the second passage by using Panasonic CCTV camera (WV-BP312E) (Panasonic) and an Olympus Microscope (CK2 ULWCD 0.3) (Olympus).

### Filum terminale culture

Cells were isolated from the tissue according to a protocol previously described by Johansson et al [12]. Connective tissue was peeled off and the FT was mechanically dissociated with scalpels and scissors and placed in a dissociation medium, consisting of 200 U/ml DNAse (Sigma-Aldrich) and 0,025% trypsin (Invitrogen) or 10 U/ml papain (Worthington) in a 37°C water bath for 30 min. Tissue was triturated three times every 5 min, and further incubated. To stop the enzymatic reaction, 10 mg/ml BSA (Sigma-Aldrich) and 10 mg/ml ovomucoid (Worthington) were mixed with L15, added and mixed with the dissociation medium. Cells were collected by centrifugation at 220 g for 5 min. To further enrich for progenitor cells, 0.9 M sucrose in Hanks' Balanced Salt Solution (HBSS) (Invitrogen) was added to the tissue solution followed by centrifugation at 750 g for 10 min and washing with L15. The cell pellet was resuspended and to allow single cell cultures in 5 or 10 cm Ø petri dishes in neurosphere medium, composed of DMEM/F12 (invitrogen), HEPES (Gibco), B27 supplement (Gibco) and Penicillin-Streptomycin (Invitrogen). To propagate human FT progenitor cells, the following growth factors were added; 20 ng/ml recombinant human epidermal growths factor (EGF, R&D systems) or mouse EGF (BD Bioscience) and 20 ng/ml recombinant human basic fibroblast growth factor (bFGF, R&D systems). After the first passage, 10 ng/ml recombinant human leukaemia inhibitory factors (Chemicon) were added to the medium. The neurospheres were cultured until they reached a critical size which normally took seven weeks. Another set of cells was cultured in neurosphere medium with the addition of 20% BIT 9500 medium. BIT medium consists of BSA, insulin (SIGMA) and 20 ng/ml transferrin (Stem Cell Technologies). Platelet-derived growth factor- BB (PDGF-BB) (R&D systems) was added at 30 ng/ml. Cells were incubated for eight days and the medium was changed three times. Cells cultured with BIT 9500 medium were differentiated on gelatine-coated plates (Sigma-Aldrich). In order to exclude contamination of the data by tumor forming cells, all tissue preparations were screened for the presence of the P53 gene. Cell cultures were fixed with 4% PFA and blocked in PBS, 0.1% saponin and 5% goat serum. Preparations were incubated with the primary antibodies; rabbit-anti-GFAP 1∶1000 (DAKO), mouse anti-h-Nestin 1∶1000(R&D Systems), mouse anti-Tubulin β-III 1∶50 (Chemicon) for 24 h at 4° C, rinsed in PBS and subsequently incubated with species-specific secondary antibodies diluted in PBS; Alexa 488 donkey anti-mouse 1∶500 (Molecular Probes/Invitrogen), Cy3 donkey anti-rabbit 1∶1000 (Jackson Immunoresearch). Cells were counterstained with nuclear markers Hoechst (Molecular Probes, Invitrogen). The tissue was mounted in Mowiol (Calbiochem, VWR International).

In order to evaluate the proliferation capacity of FT another set of culture was performed as previously described. After passage the neurosphers were pulsed with 5-bromo-2’-deoxyuridine (BrdU, Sigma) for 48 h in the presence of EGF and bFGF. The spheres were placed on coated glasses and before fixating them with PFA 4%. The glasses were stained for BrdU, Sox2 and DAPI (data not shown). BrdU incorporation was detected using rat anti-BrdU (1∶50, AbD, Serotec) and revealed by secondary antibody goat anti-rat IgG Alexa 488 (1∶100, Invitrogen).

Statistical analysis was performed by using unpaired two-tailed Students *t*−test (GraphPad Prism® 5.0, GraphPad Software Inc., La Jolla, CA, USA). Data are presented as mean ± SD, and *P <0.05 was defined as statistical significance.

### Ethical aspects

This study was carried out in accordance with the Helsinki declaration. The experimental procedures were approved by the Stockholm county ethical committee for human research (app no 138–31). It is emphasized that the human tissue used for these experiments should otherwise have been discarded. Tissue from the patients was only obtained from Karolinska University Hospital, and under informed consent by the patient or parent in accordance with the approval by Stockholm county ethical committee at Karolinska University Hospital. Information was given verbally and in writing. A verbal consent, as stated in the patients records was in this cases in agreement with the law of biobanking. The animal experiments were approved by the Stockholm county ethical committee (approval no 45–07) Animal care was in accordance to the recommendations at Karolinska Institutet.

In summary, our findings add further evidence for the existence of cells with neuronal progenitor nature within the FT. We demonstrate the abundance and distribution of the cells and their ability to respond to growth factors. We also demonstrate that the progenitor cells detected from young patients have greater growth capacity, higher proliferative ability and more easy to expand than cells derived from older donors. Since the FT can be removed without functional impairment it may represent an attractive CNS reservoir of neural stem/progenitor cells.
